# Contribution and expression of renal drug transporters in renal cell carcinoma

**DOI:** 10.3389/fphar.2024.1466877

**Published:** 2025-02-17

**Authors:** Yawen Zuo, Tong Li, Shilei Yang, Xuyang Chen, Xufeng Tao, Deshi Dong, Fang Liu, Yanna Zhu

**Affiliations:** ^1^ Department of Pharmacy, First Affiliated Hospital of Dalian Medical University, Dalian, China; ^2^ Department of Medical Oncology, The Second Affiliated Hospital of Dalian Medical University, Dalian, Liaoning, China

**Keywords:** renal cell carcinoma, renal tubular epithelial cell, drug transporters, therapeutic effect, adverse reaction

## Abstract

Renal cell carcinoma (RCC) is a common substantive tumor. According to incomplete statistics, RCC incidence accounts for approximately 90% of renal malignant tumors, and is the second most prevalent major malignant tumor in the genitourinary system, following bladder cancer. Only 10%–15% of chemotherapy regimens for metastatic renal cell carcinoma (mRCC) are effective, and mRCC has a high mortality. Drug transporters are proteins located on the cell membrane that are responsible for the absorption, distribution, and excretion of drugs. Lots of drug transporters are expressed in the kidneys. Changes in carrier function weaken balance, cause disease, or modify the effectiveness of drug treatment. The changes in expression of these transporters during cancer pathology results in multi-drug resistance to cancer chemotherapy. In the treatment of RCC, the study of drug transporters helps to optimize treatment regimens, improve therapeutic effects, and reduce drug side effects. In this review, we summarize advances in the role of renal drug transporters in the genesis, progression, and treatment of RCC.

## 1 Introduction

Kidney cancer is a relatively common type of cancer, accounting for approximately 3%–5% of all malignancies (Rose and Kim, 2024). Renal cell carcinoma (RCC) is a type of cancer that originates from the tubular epithelial cells of the kidney, and it has three histological subtypes: clear cell RCC (ccRCC), papillary RCC (pRCC), and chromophobe RCC (chRCC) (Hosseiniyan Khatibi et al., 2022). RCC represents over 90% of all kidney cancer (Hsieh et al., 2017). Moreover, RCC incidence is increasing worldwide, with higher rates observed in developed than in developing countries (Padala and Kallam, 2023). Although overall RCC incidence has increased over the last three decades, the death rate of RCC has declined rapidly because of early diagnosis and treatment (Medina-Rico et al., 2018). Even with progress in disease control, some patients may still develop locally advanced diseases and distant metastases (Vasudev et al., 2020; Ciarimboli et al., 2021). The field of RCC treatment has significantly changed over the past three decades. Indeed, the treatment landscape for RCC has undergone significant transformation in recent years owing to steady progress in the development of targeted therapeutics (Pérez-Herrero and Fernández-Medarde, 2015) and immunotherapy (Szeto and Finley, 2019). Immune checkpoint inhibitors (ICI) in combination with vascular endothelial growth factor tyrosine kinase inhibitors (TKI) have become the standard primary therapy for many advanced RCC (Chen et al., 2023).

Drug transporters are responsible for the absorption, distribution, metabolism, and excretion of drugs from the human body. The relationship between RCC and various transporters involves many aspects such as drug metabolism, nutrient transport, and cell survival. Therefore, transporters play a crucial role in sustaining the physiological balance of the body and in administering drugs. Changes in the function of transporters can impair homeostasis, cause disease, or modify the efficacy of the drugs (Ciarimboli, 2023). Although many renal drug transporters have been characterized in detail with respect to the significance for proper kidney function, their role in kidney cancer progression is less known. Drug transporter expression may reflect resistance to systemic therapy in RCC, and can be used to predict prognosis. This review summarizes progress in the significance of renal drug transporters in the genesis, progression and treatment of RCC.

## 2 Types of drug transporters predominantly expressed in the kidney

### 2.1 Classification of drug transporters

Drug transporters are typically classified into two major families: the solute carrier (SLC) family and the adenosine triphosphate-binding cassette (ABC) family (Liu, 2019a).

#### 2.1.1 ABC family

The largest transporter family is the ABC family. The ABC transporter family is one of the most diverse groups of transmembrane proteins involved in active transport processes. The ABC proteins have numerous functions to list in detail. However, they mainly transport a diverse range of substrates, from simple ions to polar, amphiphilic, and hydrophobic organic molecules, peptides, complex lipids, and even small proteins (Theodoulou and Kerr, 2015). Over 40 ABC transporters have been discovered in humans and partitioned into seven subfamilies (ABCA to ABCG) based on various criteria such as gene structure and amino acid sequence. At least 11 ABC transporters have been implicated in multi-drug resistance, including P-glycoproteins (P-gp/*ABCB1*), multi-drug resistance proteins (MRP/*ABCC*), and breast cancer resistance proteins (BCRP/*ABCG2*). They actively remove anti-tumor drugs from cancer cells, reduce their intracellular concentrations, thereby conferring resistance to chemotherapy (Liu, 2019b). These ABC transporters have significant effects on many cell types, including renal tubular cells. The reabsorption and secretion functions of the nephron are mediated by a variety of transporters located in the basolateral and luminal membranes of the tubular cells. Many studies indicated that transporters play important roles in drug pharmacokinetics and demonstrated the impact of renal transporters on the disposition of drugs, drug-drug interactions (DDI), and nephron toxicities (Yang and Han, 2019).

#### 2.1.2 SLC family

The SLC transporters include the *SLC-21A* gene subfamily (organic anion transporter polypeptide, OATP), *SLC-22A* gene subfamily (organic anion transporter, OAT; organic cation transporter, OCT; organic cation/carnitine transporter, OCTN), *SLC-15A* gene subfamily (peptide transporter, PEPT), and *SLC-47A* gene subfamily (multi-drug and toxin excretion, MATE) (Liu, 2019c). OCTs and OCTNs are responsible for transporting organic cations, and are involved in the transport of several drugs in the body. OATP-4C1 is the major OATP transporter in kidney, mainly located on the basolateral membrane of proximal renal tubular cells (Sato et al., 2017).

### 2.2 Drug transporters predominantly expressed in the kidneys

The kidney performs the critical task of maintaining homeostasis through the coordinated action of multiple transport systems specifically expressed in different parts of the kidney’s functional unit, the nephrons (Lee et al., 2015). Exogenous substances secreted by the kidney mainly occur in the proximal renal tubules, which have specific transport mechanisms that facilitate the passage of foreign substances from the blood into the tubular cells (uptake) and from these cells into the tubular fluid (excretion).

**FIGURE 1:**
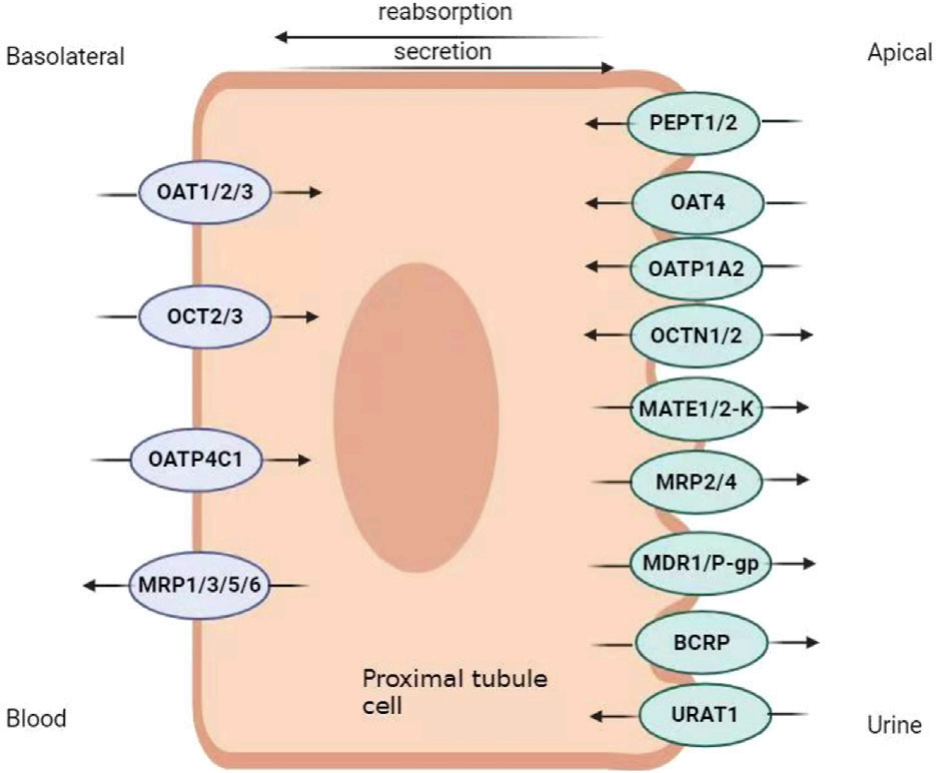
Renal transporter distribution map. OAT, organic anion transporter; OCT, organic cation transporter; OATP, organic anion transporter polypeptide; MRP, multi-drug resistance protein; OCTN, organic cation/carnitine transporter; PEPT, peptide transporter; MATE, multi-drug and toxin excretion; URAT, urate transporter.

Transporters in renal proximal tubule epithelial cells (RPTEC) contribute to drug disposition. In the proximal renal tubules, epithelial cells have two distinct membrane domains, the basal-lateral membrane and the apical (or lumen) membrane, both of which have transporters. Basolateral transporters are responsible for absorbing solutes from the blood into the epithelium, whereas apical transporters are responsible for excreting solutes from the cell into tubular fluid (Ivanyuk et al., 2017). Drug transporters are divided into uptake and efflux transporters, according to the direction of transmembrane transport of the substrates (Figure 1).

#### 2.2.1 Uptake transporters

The basolateral uptake of drugs by transporters OAT1, OAT3, OATP-4C1, OCT2, and OCT3 is critical for the kidneys to process a variety of drugs and exogenous substances and, ingested substrates at target sites for efficacy. OATs are instrumental in the tubular secretion of numerous drugs, specifically antibiotics, antiviral therapeutics, diuretics, and non-steroidal anti-inflammatory drugs (Momper et al., 2019). OAT1 and OAT3 are the most studied SLC families (Nigam, 2018). OAT1 is located mainly in the basolateral membrane of proximal tubular cells (Nigam, 2015). OAT2 binds specifically to antiviral medications (Nigam, 2015), and OAT3 is the most abundantly expressed transporter in the proximal tubules of the human kidney (Bunprajun et al., 2019). OAT1 and OAT3 transport penicillin and non-steroidal anti-inflammatory drugs (Koepsell et al., 2007; Dudley et al., 2000; Bourdet et al., 2005; Jung et al., 2008; Sato et al., 2008; Tahara et al., 2005). OAT substrates include anti-tumor drugs methotrexate and ubenimex (Zhu et al., 2014). In addition, OAT4 and urate transporter (URAT) 1, both of which belong to the solute vector family, are expressed in the apical membranes of proximal renal tubular cells. OAT4 and URAT1 facilitate the reabsorption of uric acid from proximal tubular cells into the blood (Xu et al., 2017). Probenecid and benzbromarone are commonly used to treat hyperuricemia. They block the reabsorption of uric acid by inhibiting URAT1, and promote urate excretion, thereby reducing blood uric acid levels (Shin et al., 2011). Moreover, the angiotensin II receptor blocker losartan also binds to URAT1, increases uric acid excretion, and reduces blood uric acid levels (Vanwert et al., 2010).

OCTs are members of the SLC22 family (Döring and Petzinger, 2014), and OCT1, OCT2 and OCT3 are expressed in humans. The main substrates of OCTs are fampridine, cisplatin, oxaliplatin, metformin, lamivudine and adolol (Cheung et al., 2017). Metformin reabsorption is influenced by OCT1, which is located on the apical membrane of both the proximal and distal tubules in the kidney (Ivanyuk et al., 2017). OCT2 mainly transports metformin, cisplatin, lamivudine and atenolol (George et al., 2017; Jung et al., 2013). OCT3 is also expressed in the kidney (George et al., 2017). OATP-4C1 is the primary carrier for the transport of digoxin, methotrexate and sitagliptin (Sato et al., 2017; Ivanyuk et al., 2017; Klaassen and Lu, 2008). OCTNs includes OCTN1 and OCTN2 (Pochini et al., 2019). OCTN1 can transport some important drugs such as verapamil, quinidine and gabapentin (Ivanyuk et al., 2017; Nigam, 2015). OCTN2 transports cefepime (Ivanyuk et al., 2017).

PEPT1 is mainly expressed in the small intestine, and its role in intestinal inflammation and inflammatory bowel disease has been previously elucidated (Ingersoll et al., 2012). PEPT2, as an apically expressed transporter, mediates the reabsorption of small anionic peptides (dipeptides and tripeptides) coupled with H^+^ uptake, and may thus influence the pharmacokinetics of various peptide-like compounds (Ivanyuk et al., 2017; Sala-Rabanal et al., 2008). It has been shown to recognize some ß-lactam antibiotics (ampicillin, amoxicillin, cephalexin, cefaclor, cefadroxil), valacyclovir, and bestatin, and is likely to mediate their reabsorption from the primitive urine, thus potentially slowing down their elimination (El-Sheikh et al., 2008; Li et al., 2006; Ganapathy et al., 1998; Tomita et al., 1990).

#### 2.2.2 Efflux transporters

Efflux transporters pump the substrate out of the cell to reduce the cellular substrate concentration. They are ABC transporters such as MPR2, MPR4, P-gp and BCRP. Although MATE proteins belong to the SLC superfamily, they also function as efflux transporters. MATE proteins include MATE 1 and MATE 2K (Veiga-Matos et al., 2020). The MATE proteins facilitate the translocation of norfloxacin, ciprofloxacin, levofloxacin, cephalexin, cefradine, dofetilide, cisplatin, oxiliplatin, nadolol, emtricitabine, metformin and cimetidine (Ivanyuk et al., 2017; Nies et al., 2016; Misaka et al., 2016; Uddin et al., 2022; Waissbluth et al., 2023; Miyamae et al., 2001; Reznicek et al., 2017; He et al., 2011). MRPs transport various substrates, including anions formed when drugs (such as methotrexate and cisplatin) conjugated with sulfate, gluconate, or glutathione (Borst et al., 2000). Urine removal is regulated by OCTN1, OCTN2, MATE1, MATE 2K, P-gp, MPR2, MPR4, and BCRP (Morrissey et al., 2013). P-gp has been extensively studied. P-gp transports a variety of anti-tumor drugs, such as paclitaxel and vincristine (Hlavata et al., 2012; Waghray and Zhang, 2018). P-gp also transports various anti-infective drugs, such as macrolides (azithromycin, erythromycin, clarithromycin) and tetracycline (Akamine et al., 2019). BCRP can actively remove anti-tumor drugs such as imatinib, methotrexate (Fletcher et al., 2010). MRP4 also affects uric acid secretion in proximal tubules (Yang et al., 2010). Methotrexate is a MRP4 substrate, which is secreted into the tubule lumen by MRP4 (Hoque et al., 2009). Multi-drug efflux pumps from different families expel antimicrobial agents from the bacteria, thereby leading to drug resistance (Chitsaz and Brown, 2017). When RCC patients require the concurrent use of antimicrobial agents, attention should be paid to transporter-mediated DDI, with a focus on the efficacy and adverse reactions of both anti-tumor drugs and antimicrobial agents. The main substrates of some drug transporters are listed in Table 1.

**TABLE 1:** Main drug transporters in the kidney and their substrates or inhibitors.

Location	Transporter	Substrate	Inhibitor	Reference
Basolateral membrane	OAT1/3	Methotrexate, Ubenimex, Tetracycline, Acyclovir	NSAIDs, Leflunomide, Cytarabine, Vincristine, Hydrocortisone, Mitoxantrone	Zhu et al. (2014), Babu et al. (2002), Liao et al. (2020), El-sheikh et al. (2013)
	OCT2/3	Fampridine, Cisplatin, Oxaliplatin, Metformin, Lamivudine, Nadolol	Fampridine, NSAIDs, Dolutegravir, Isavuconazole, Duloxetine, Cetirizine	Jung et al. (2013), Misaka et al. (2016), Koepsell (2013), Mulgaonkar et al. (2013), Khamdang et al. (2002), Xiao et al. (2018), Nepal et al. (2022), Noguchi et al. (2017)
	OATP-4C1	Remdesivir, Digoxin, Methotrexate	Digoxin, Ouabain	Yamaguchi et al. (2010), Sato et al. (2021), Mikkaichi et al. (2004)
Apical membrane	P-gp/MDR1 (*ABCB1*)	Erythromycin, Clarithromycin, Azithromycin Tetracycline, Digoxin, Methotrexate, Pazopanib, Sorafenib, Everolimus, Tisirolimus, Paclitaxel, Gefitinib, Vincristine, Nadolol, Elacridar	Isavuconazole, Axitinib, Cabozantinib, Everolimus, Verapamil, Cyclosporine, Erythromycin, Ritonavir, Ketoconazole, Quinidine, Diltiazem	Misaka et al. (2016), Akamine et al. (2019), Rengelshausen et al. (2003), Banerjee et al. (2000), Milane et al. (2007), Perez-Tomas (2006), EL-Mahdy et al. (2020), Dash et al. (2017)
	BCRP (*ABCG2*)	Methotrexate, Sorafenib, Doxorubicin, Mitoxantrone, Docetaxel, Gefitinib,5-Fluorouracil, Imatinib	Isavuconazole TKI (e.g., Gefitinib, sunitinib), Elacridar	Reustle et al. (2018), Safar et al. (2019), Yanase et al. (2004), Alves et al. (2022), Sun et al. (2022)
	MRP2/4	Cisplatin, Methotrexate, Anthracyclines, Vinca alkaloids, Epipodophyllotoxins, Paclitaxel, Acyclovir, Fosinopril	Leflunomide, Cyclophosphamide, Mydrocortisone, Tacrolimus, Cyclosporine, Vincristine, Vincristine, 6-mercaptopurine	Liao et al. (2020), El-Sheikh et al. (2013), Pedersen et al. (2017), Green and Bain (2013)
	MATE1/2K	Dofetilide, Cisplatin, Oxiliplatin, Cimetidine, Metformin, Norfloxacin, Ciprofloxacin, Levofloxacin, Nadolol, Emtricitabine	Dolutegravir, Famotidine, Cimetidine, Pyrimethamine	Misaka et al. (2016), Uddin et al. (2022), Waissbluth et al. (2023), Miyamae et al. (2001), Reznicek et al. (2017), He et al. (2011)
	OAT4	Estrone sulfate, Urate, Ibuprofen, Indomethacin, Ketoprofen, Salicylate, Olmesartan, Levocetirizine	Candesartan, Siladiate, Losartan, Valsartan, Tranilast	Nigam (2015), Khamdang et al. (2002), Noguchi et al. (2017), Burckhardt and Burckhardt (2011), Noguchi et al. (2021), Yamashita et al. (2006), Mandal et al. (2017)
	OCTN1/2	Etoposide, Oxaliplatin, Imatinib	Cetirizine, Quinidine,Tetracycline, Minocycline	Hu et al. (2012), Jong et al. (2011), Hu et al. (2008)
	PEPT2	β-lactam antibiotics, Enalapril; valacyclovir; bestatin		Döring and Petzinger (2014), El-Sheikh et al. (2008), Li et al. (2006), Ganapathy et al. (1998), Tomita et al. (1990), Inui et al. (2000), Smeets et al. (2020)

ABC, adenosine triphosphate-binding cassette; P-gp, P-glycoproteins; MRP, multi-drug resistance protein; OATP, organic anion transporter polypeptide; OAT, organic anion transporter; OCT, organic cation transporter; OCTN, organic cation/carnitine transporter; PEPT, peptide transporter; MATE, multi-drug and toxin excretion; BCRP, breast cancer resistance protein; NSAID, non-steroidal anti-inflammatory drugs; TKI, tyrosine kinase inhibitor.

## 3 Changes in expression of transporters influence the occurrence, development, and treatment of RCC

Under normal physiological conditions, the expression and regulation of drug transporters can help maintain kidney homeostasis (Caetano-pinto et al., 2022). The activity and the expression of drug transporters plays a key role in renal secretion and reabsorption function in RPTEC. Moreover, the expression of drug transporters in RCC cells often differ from those in normal renal cells (Table 2). In RCC, changes in transporter expression affect uptake and efflux processes of anti-tumor drugs, thus affect the therapeutic effects of anti-tumor drugs. The expression and function of drug transporters are influenced by many factors, such as microbiota influence, post-translational modification, transcriptional regulation, enriched epigenetic regulations and exogenous modulations (Yin et al., 2024). Two cellular regulatory processes contribute to the pathophysiology of RCC: DNA methylation (Herman et al., 1994) and epidermal growth factor receptor (EGFR) signaling (Minner et al., 2012; Muroni et al., 2021). A hypermethylated state is associated with the loss of the Von Hippel-Lindau tumor suppressor protein (Clifford et al., 1998) and deregulation of enzymes and carrier proteins responsible for drug metabolism and disposition, including drug transporters (Winter et al., 2016). Epigenetic changes in drug transporter genes are associated with drug response in cancer (Ivanov et al., 2012), and multiple types of epithelial cancers are associated with defective, overexpressed, or constitutive activation of EGFR (Uribe et al., 2021).

**TABLE 2:** Changes in renal transporters during RCC.

Tissue or cell	Transporter	Change	Reference
Kidney tissue in patients with RCC	P-gp	Upregulation	Walsh et al. (2009)
786-O cells	P-gp	Upregulation	Sato et al. (2015)
Kidney tumor cell lines 786-O, RCCNG1, A498, LN78, and ACHN	OAT1	Downregulation	Shnitsar et al. (2009)
Kidney tumor cell lines A498 and 786-O	OCT3	Upregulation	Shnitsar et al. (2009)
RCC cell lines CAKI-1	OCT2	Downregulation	Lee et al. (2017)
Kidney tissue in patients with primary pRCC, primary ccRCC	OCT2	Downregulation	Visentin et al. (2018)
ccRCC metastasis tissue	OCT2	Upregulation	Visentin et al. (2018)
786-O, 769-P, HEK-293 cell lines	OCT2	Downregulation	Chen et al. (2019a)
five RCC cell lines (Caki-1, Caki-2, A-498, ACHN, and 786-O)	OCT2	Downregulation	Winter et al. (2016)
RCC tissues	OCT2	Downregulation	Liu et al. (2016)
Kidney tissue in patients with ccRCC	BCRP	Upregulation	Lee et al. (2017), Reustle et al. (2018)
Japanese RCC patients with the rs2231142 C421A genetic variant	BCRP	Downregulation	Low et al. (2016)
Kidney tissue in patients with RCC	MRP1/3/4	Upregulation	Rhodes et al. (2004)
Kidney tissue in patients with ccRCC	MRP2	Upregulation	Lee et al. (2017)
Tumor tissue and normal kidney tissue from patients with ccRCC	MRP2	Upregulation	Schaub et al. (1999)

RCC, renal cell carcinoma; pRCC, papillary RCC; ccRCC, clear cell RCC; P-gp, P-glycoproteins; MRP, multi-drug resistance protein; OAT, organic anion transporter; OCT, organic cation transporter; BCRP, breast cancer resistance protein.

### 3.1 SLC family

The urea transporter encoded by *SLC14A1* (UT-B) plays a key role in the kidney, where it transports urea and maintains normal kidney function. Mutations or abnormal expression of *SLC14A1* may be associated with the occurrence and development of kidney cancer. *SLC14A1* is expressed at lower levels in kidney cancer tissues than in normal kidney tissues (Wan et al., 2023). Moreover, the higher the *SLC14A1* expression levels, the lower the kidney cancer differentiation grade and the higher the overall patient survival rate. This indicates that *SLC14A1* inhibits the occurrence and development of kidney cancer (Wan et al., 2023). Therefore, *SLC14A1* is a potential target for the treatment of kidney cancer. However, these hypotheses are still preliminary and further research is needed to confirm the exact relationship between *SLC14A1* and kidney cancer. In addition, the occurrence and development of kidney cancer is complex processes involving the interaction of multiple genes and factors; therefore, *SLC14A1* cannot be regarded as the sole cause of kidney cancer.

Sodium-coupled dicarboxylate transporter (NaDC1) encoded by *SLC13A2* plays an important role in regulating the acid-base balance, preventing calcium kidney stones, regulating sodium-chloride transport in the collecting duct, and regulating blood pressure (Osis et al., 2019). Apical NaDC1 immunomarker is present throughout the proximal convoluted tubule but is not detected in kidney tumors, including ccRCC and pRCC, that presumably originate in the proximal convoluted tubule, as well as in tumors of non-proximal convoluted tubule origin (Lee et al., 2017). This suggests that NaDC1 expression may is downregulated in RCC.

The relationship between the expression of SLC22 genes and survival in patients with kidney cancer was assessed. In the Cancer Genome Analysis (TCGA) project, two RCC RNA-seq datasets, namely ccRCC and pRCC, were found to have multiple differentially expressed (DE) SLC22 transporter genes compared with those in normal kidney tissue. These included *SLC22A6*, *SLC22A7*, *SLC22A8*, *SLC22A12*, and *SLC22A13*. The patients with disease had an association between overall survival and expression of most of these DE genes. Many important SLC22 genes, including those of the OAT and OAT-related groups, had decreased expression over the continuum of stages of RCC from well-functioning, healthy kidneys to advanced metastatic disease. Alternatively, analysis of patients with different classifications of tumor size/progression, lymph node involvement, and presence of metastasis identified multiple SLC22 transporters as significantly changed, often decreasing with severity. A number of the identified transporters (e.g., URAT1/*SLC22A12*, OAT1/*SLC22A6*, OAT3/*SLC22A8*, BCRP/*ABCG2*) are well-established uric acid transporters. This may be clinically important since, a number of studies indicate that altered uric acid levels and kidney cancer are associated (Whisenant and Nigam, 2022).

According to the Oncomine cancer transcriptome database, most uptake transporters except OCTN2 and PEPT1 are transcriptionally repressed in RCC (Rhodes et al., 2004), and the kidneys contain various SLC22 transporters (Rosenthal et al., 2019; Nigam et al., 2015). In RCC, most of the genes for SLC22 are downregulated, which affects the uptake of some anti-tumor drugs in the kidneys, thereby impacting the therapeutic efficacy of these drugs and potentially leading to the progression of the cancer. Winter et al. (2016) found that, OCT2 expression in RCC cells was below the limit of quantification. Western blot analysis revealed no OCT2 protein expression and OCT expression was restored by inhibiting its methylation. Oxaliplatin, a platinum-based anticancer drug, covalently binds to DNA to form DNA adducts, which trigger various signal transduction pathways. Platinum resistance is caused by insufficient DNA-binding; thus, cellular accumulation of the drug is an important determinant of oxaliplatin’s cytotoxicity (Kelland, 2007). Early clinical trials have shown that oxaliplatin is ineffective against advanced RCC (Chaouche et al., 2000; Porta et al., 2004). Furthermore, OCT2 is a major transporter that enhances cellular uptake and cytotoxicity of oxaliplatin *in vitro* (Tatsumi et al., 2014). Most proteins showing reduced expression have not yet been characterized; however, studies strongly suggest that reduction in uptake transporters contributes to multidrug resistance in RCC (Puris et al., 2023).

The expression of OCT2 in RCC is has been relatively well studied. Caetano-pinto et al. (2022) (Lee et al., 2017) characterized the activity, expression, and potential regulatory mechanisms of renal drug transporters in RCC *in vitro* using different cell lines and a non-malignant RPTEC. They found that the expression of OCT2 was absent in the RCC cell line, CAKI-1. Moreover, a limited amount of OCT2 expression was recovered by the inhibition of methylation in CAKI-1 cells. Hence, both OCT2 and MATE 2K are repressed in RCC cells, resulting in insufficient accumulation of oxaliplatin and subsequent therapy failure. In RCC cell lines, decitabine (DAC) was used to inhibit DNA methylation by blocking DNA methyltransferases. OCT2 but not MATE-2K expression was restored in RCC cells after DAC treatment, resulting in high oxaliplatin uptake and low oxaliplatin efflux, high oxaliplatin accumulation, and increased oxaliplatin cytotoxicity (Liu et al., 2016). Therefore, sequential combination of DAC and oxaliplatin is a promising treatment option to sensitize RCC cells to oxaliplatin by activating OCT2-mediated transport.

The effect of microRNAs on transporters has also been studied in RCC. MicroRNAs (miRNA) are a set of endogenous single-stranded small RNAs with a length of approximately 21–23 nt, they modify gene expression post-transcriptionally, participate in the mediation of over 60% of protein-coding gene expression, and participate in almost all intracellular biological processes. Hence, miRNAs not only affect normal cell growth, differentiation, and other aspects, but also play a role in cancer, heart disease, inflammation, and more (Shen et al., 2012; Lu et al., 2017). With the growth of miRNA research and the development of molecular biology technology, an increasing number of studies have shown that abnormal expression of miRNAs can affect the tumor formation and growth. The miRNA expression profiles in RCC tissue samples have been screened and have revealed that the formation and metastasis of renal cancer are strongly correlated with some miRNAs (Miranda-Poma et al., 2022; Petillo et al., 2009). Moreover, miRNAs belonging to the Let-7 family are significantly downregulated in patients with nephroblastoma (Huo et al., 2010). Most miRNAs in RCC tissues exhibited a downward trend. These abnormally expressed miRNAs can be used as targets or targeted drug components to inhibit their downstream regulation to curb tumor proliferation and progression. They can also be used as biomarkers for the diagnosis of RCC before radioactive examination. High miR-630 levels inhibit the expression of OCT2 mRNA, thereby inhibiting its protein expression levels and weakening its uptake of classical substrates and the anticancer drug oxaliplatin (Chen et al., 2019a). This suggests that the inhibition of OCT2 as a result of high miR-630 expression is one of the mechanisms of oxaliplatin resistance in RCC.

OCT2 was also differentially expressed in primary and metastatic tissues of kidney cancer. Interestingly, a significant decrease in OCT2 mRNA expression was found in primary RCC but not in metastatic RCC (Visentin et al., 2018). Moreover, the main choline transporter in the kidney, OCT2, recognizes fluorocholine as a substrate (Visentin et al., 2017). Furthermore, a high likelihood exists for the dominant role of OCT2 in [^18^F] fluorocholine renal uptake, and changes in OCT2 expression levels during renal carcinogenesis may affect [^18^F] fluorocholine accumulation. Compared with that in surrounding normal tissues, metastatic RCCs may accumulate abnormal amounts of [^18^F] fluorocholine due to OCT2 modulation. The use of [^18^F] fluorocholine positron emission tomography/computed tomography may improve sensitivity for the detection of early-stage metastatic disease, which is a major clinical challenge during the initial staging of RCC (Chaouche et al., 2000).

*SLC22A3* (human OCT3) is highly expressed in two of the five RCC cell lines (A498 and 786-O) (Walsh et al., 2009). In A498 cells, [^3^H]MPP (the model substrate of OCT3) accumulation was >10 fold higher than in ACHN cells. Irinotecan, vincristine, and melphalan inhibited uptake of [^3^H]MPP into these cells and also into hOCT3 stably transfected Chinese hamster ovary (CHO) cells. The growth of CHO-hOCT3 was inhibited by 20% more with irinotecan and by 50% more with vincristine compared with non-transfected CHO cells. Melphalan produced 20%–30% more inhibition in hOCT3-expressing cells compared with non-expressing control cells. Expression of hOCT3 in kidney carcinoma cell lines increases chemosensitivity to melphalan, irinotecan, and vincristine. That supports the hypothesis that the sensitivity of tumor cells to chemotherapeutic treatment depends on the expression of transporter proteins mediating specific drug accumulation into target cells. This fact renders OCT3 an appropriate candidate for individualized kidney tumor therapy (Shnitsar et al., 2009). Along these lines, it is worthwhile considering to test for OCT3 expression and to tailor the cytostatic therapy.

Little was known about the expression of OATs in kidney tumors and their interactions with cytostatics. The expression of SLC transporters in the kidney tumor cell lines 786-O, RCCNG1, A498, LN78, and ACHN, and their interactions with chemotherapeutics have been investigated (Walsh et al., 2009). An mRNA level analysis in kidney cancer cell lines revealed the presence of OAT1. However, the uptake of PAH was relatively low, and it was not inhibited by 500 μmol/L probenecid (a standard blocker of OATs). OATs are, therefore, unsuitable as targets for anionic cytostatic chemotherapy for RCC.

### 3.2 ABC family

Overexpression of ABC transporters in cancer cells is a well-documented multi-drug resistance mechanism. The transport of drugs from the intracellular to the extracellular is facilitated by various transporters, which the expression and function of those transporters are highly regulated (König et al., 2013). In addition, hyperexpression of these transporters has been reported in untreated solid tumors and various types of leukemia (Nakanishi and Ross, 2012). However, the development of such inhibitors has been challenging owing to the specificity and complexity of the function of ABC transporters. Therefore, the evolution of resistance to multiple drugs in cancer cells is a significant barrier for the successful treatment of the disease (Wu et al., 2023).

Several diseases, including cancer, are affected by the inter-individual variability in BCRP/*ABCG2*. BCRP expression may be reduced by the minor alleles of two *ABCG2* variants, rs2231137 G34A (V12M) and rs2231142 C421A (Q141K). Gene variants rs2231137 G34A and rs2231142 C421A have been reported to be associated with disease risk, reduced efficacy of drug treatments, and increased adverse reactions in different human diseases (Chen et al., 2019b). Relationship between carcinogenesis and common ABCG2 variants is controversial in population-based association studies in various types of cancer. Association of ABCG2 rs2231142 C421A with the development of breast cancer was examined in 100 Kurdish patients and 200 healthy controls (Ghafouri et al., 2016). Patients with AA genotype of rs2231142 were at a higher risk of breast cancer. A meta-analysis found that the rs2231142 A allele is associated with a lower risk for the development of multiple cancer types, including leukemia and colorectal cancer. The relationship between the common ABCG2 variants and cancer risk is complex and may be different in divergent human populations and variable across cancer types. Further studies are needed to clarify the impacts of BCRP transporter genotypes upon carcinogenesis. However, the common ABCG2 variants may also be related to severe drug-induced adverse reactions to chemotherapy. In a study of 219 Japanese patients with RCC, the rs2231142 C421A genetic variant was associated with severe thrombocytopenia following sunitinib therapy (Low et al., 2016). Therefore, sunitinib doses must be adjusted in patients with the rs2231142 A variant.

ABCC2 belongs to the ABC transporter family and induced chemotherapy resistance; hence, it was named MRP2 (Jeong et al., 2015). Reportedly, the MRP 2 and other ABC transporters influence the anti-tumor therapeutic effects of TKIs (Kathawala et al., 2015; Shibayama et al., 2011). As a TKI, sunitinib disrupts signaling pathways that lead to tumor proliferation and angiogenesis in cancer cells and is often considered the frontline treatment for pRCC. Moreover, as a drug transporter, MRP2 may influence the effect of sunitinib on cancer cells (Warta et al., 2014; Zhang et al., 2014). The development of drug resistance is a common obstacle for TKI treatment. One hypothesized resistance mechanism is the active expulsion of intracellular substances by ABC transporter proteins (He and Wei, 2012). Therefore, a combination of sunitinib and MRP2 blockers for the treatment of pRCC2 may enhance anticancer efficacy of Sunitinib. Saleeb et al. conducted experiments using AKI-2 cells *in vitro* and mouse models *in vivo*. Five groups were tested: anti-vascular endothelial growth factor (sunitinib), MRP2 blocker (MK 571), mammalian target of rapamycin inhibitor (everolimus), and sunitinib + MK 571. Compared with that of the other treatment groups, the sunitinib + MRP2 blocker group produced a marked therapeutic reaction *in vitro* and *in vivo*. The MRP2 blocking results of both *in vitro* and *in vivo* experiments showed elevated sunitinib uptake levels. This suggests that the combination of sunitinib and MRP2 blockers targeting pRCC has a therapeutic potential (Saleeb et al., 2018).

Many clinical studies have evaluated the role of P-gp in the development of RCC. P-gp is an important membrane transporter that effluxes drugs from cells, and affects cellular drug concentrations, and exerts antitumor effects (Pilotto Heming et al., 2022). Lee and Thevenod (2019) found that oncogenic pituitary homeobox 2, a *de facto* master regulator of developmental organ asymmetry, upregulates the expression of P-gp in A498 RCC cells. Many anticancer drugs are the substrate of P-gp (Dei et al., 2019). Therefore, exploring the role of P-gp in RCC progression is important for improving RCC treatment outcomes. Elevated P-gp expression in RCC cells expels anticancer medications from cells, thereby resulting in decreased intracellular drug levels and subsequent diminished efficacy against tumors (Walsh et al., 2009). *ABCB1* methylation is associated with P-gp expression in RCC (especially ccRCC) (Yan et al., 2019), and the P-gp mRNA expression levels in ccRCC is higher than that in healthy kidney tissues (Yamaguchi et al., 2010). P-gp inhibition increases the anti-tumor effects of sunitinib in RCC treated with elacridar (Sato et al., 2015). In addition, both P-gp and BCRP expression were increased in patients with ccRCC compared with that in patients with normal kidney tissue or function (Reustle et al., 2018). Higher BCRP inhibition was associated with better results when sunitinib was used for cancer treatment (Reustle et al., 2018).

## 4 Future trends and research directions

Studies on the effects of drug transporters on RCC are relatively scarce, yet hold significant importance. As mentioned above, transporters are closely related to the occurrence, development, and drug efficacy of RCC. Timely RCC diagnosis and inhibition of disease progression can be achieved by exploring the expression of relevant transporters. However, RCC sometimes develop multidrug resistance to drugs, and transporters may have a vital impact on this process. Drug resistance and metastasis of malignant tumors are a key cause of death in patients with cancer and are a major challenge for cancer treatment (Jolly et al., 2019). The vast majority of cancer deaths can be attributed to the development of drug resistance (Bukowski et al., 2020); hence, drug resistance remains a major barrier to achieving a successful cure for cancer (Vasan et al., 2019). By leveraging the DDI mechanisms mediated by drug transporters and combining the use of efflux transporter inhibitors such as P-gp, BCRP, and MRP2 inhibitors. The multi-drug resistance in RCC can be reversed in some situations, because of that higher expression of efflux transporters is one of the multi-drug resistance mechanisms, there are also other mechanisms causing multi-drug resistance. Therefore, more studies on the genetic polymorphisms of drug transporters should be investigated to reveal the impact of these differences on kidney cancer treatments. However, insufficient research on the genetic polymorphism of drug transporters has been conducted. Overall, research on drug transport and kidney cancer aims to improve drug efficacy, reduce side effects, and promote personalized therapy.

## 5 Conclusion

Transporters may directly and indirectly affect the development and progression of RCC. The expression and function of these drug transporters has an important effect on drug concentrations, and the alteration of drug exposure. That may affect the efficacy and toxicity of anti-tumor drugs. Thus, current information indicates that the changes of transporters have indirect affected disease occurrence or progression. Understanding the role of these drug transporters in RCC will provide more information about specific treatments. Researchers and clinicians can consider these factors in order to choose a suitable therapeutic drug or a combination drug strategy to maximize the concentration of the drug in the tumor, improve the efficiency of treatment and possibly increase drug resistance through combination drugs and other measures. The directly relationship between RCC and drug transporters is known less and is still worth being studied further. Hence, this review summarizes the existing literature, aims to provide support for clinical work and basic scientific research, and encourages the scientific community to focus on changes in drug transport expression to ensure the effectiveness and safety of patient medications.

## Abbreviations

RCC, renal cell carcinoma; ccRCC, clear cell RCC; pRCC, papillary RCC; ICI, checkpoint inhibitors; TKI, tyrosine kinase inhibitor; SLC, solute carrier; ABC, adenosine triphosphate-binding cassette; P-gp, P-glycoproteins; MRP, multi-drug resistance protein; OATP, organic anion transporter polypeptide; OAT, organic anion transporter; OCT, organic cation transporter; OCTN, organic cation/carnitine transporter; PEPT, peptide transporter; MATE, multi-drug and toxin excretion; BCRP, breast cancer resistance protein; RPTEC, renal proximal tubule epithelial cells; URAT, urate transporter; EGFR, epidermal growth factor receptor; NaDC1, sodium-coupled dicarboxylate transporter; DAC, decitabine; miRNA, microRNA; DDI, drug-drug interactions.
